# Contribution to the genus *Xanthocorus* Miyatake (Coleoptera, Coccinellidae, Chilocorini)

**DOI:** 10.3897/zookeys.511.9584

**Published:** 2015-07-02

**Authors:** Wenjing Li, Xiaosheng Chen, Xingmin Wang, Shunxiang Ren

**Affiliations:** 1 Engineering Research Center of Biological Control, Ministry of Education, College of Natural Resources and Environment, South China Agricultural University, Guangzhou, 510642, China

**Keywords:** Coleoptera, Coccinellidae, *Xanthocorus*, new species, China

## Abstract

The genus *Xanthocorus* Miyatake, 1970 consists of three species from China, including two new species described here: *Xanthocorus nigrosuturalis* **sp. n.** and *Xanthocorus mucronatus* **sp. n.** A key to identification of species is given. Diagnoses, detailed descriptions, illustrations, and distributions are provided.

## Introduction

*Xanthocorus* Miyatake, 1970 is a small genus within the tribe Chilocorini which currently consists of 26 genera (Łączyński and Tomaszewska 2012). Initially Miyatake (1970) treated *Xanthocorus* as the subgenus of *Exochomus*, with Exochomus (Xanthocorus) nigromarginatus Miyatake, 1970 as the type species. Kovář (1997) comprehensively revised the genera *Brumus* Mulsant and *Exochomus* Redtenbacher, and considered *Xanthocorus* Miyatake as a distinct genus. Kovář’s (1997) generic concept of *Xanthocorus* was accepted by Fürsch (2007) and is followed in the present paper.

The genus *Xanthocorus* was monotypic until recently. It was only recorded from China (Miyatake 1970; Pang and Mao 1979; Pang et al. 2004; Ren et al. 2009) and without any new species added in recent decades. In this paper two new species of *Xanthocorus* from China are described bringing to three, the number of known species in the genus. A diagnosis of the genus and a key to its known species are also given.

## Material and methods

Specimens examined in this study were collected in China. Type specimens designated in the present paper are deposited at the Department of Entomology, South China Agriculture University (SCAU), Guangzhou, and the Institute of Zoology (IOZ), Chinese Academy of Science, Beijing.

External morphology was observed with a dissecting stereoscope (SteREO Discovery V20). The following measurements were made with an ocular micrometer: TL–total length, length from apical margin of clypeus to apex of elytra; TW–total width, width across both elytra at widest part; TH–total height, from the highest part of the beetle to elytral outer margins; HW–head width in frontal view, head widest part; PL–pronotal length from the middle of anterior margin to the base of pronotum; PW–pronotal width at widest part; EL–elytral length, from the apex to the base including the scutellum; EW–elytral width, equal in TW. Morphological terms of the Coccinellidae follow Ślipiński (2007) and Ślipiński and Tomaszewska (2010).

Male and female genitalia were dissected, cleared in 10% solution of NaOH by boiling for several minutes, and examined with an Olympus BX51 microscope. Photographs of the morphological characters of the genitalia were generated with digital cameras (AxioCam HRc and Coolsnap-Procf & CRI Micro*Color), attached to microscopes using AxioVision Rel. 4.8 and Image-Pro Plus 6.0 to capture images, and photographs were cleaned up and laid out in plates with Adobe Photoshop CS 8.0.

## Taxonomy

### Xanthocorus

Taxon classificationAnimaliaColeopteraCoccinellidae

Miyatake, 1970

Exochomus (Xanthocorus) Miyatake, 1970: 312. Type species: Exochomus (Xanthocorus) nigromarginatus Miyatake, 1970, by original designation. Validated by Kovář 1997: 24.

#### Diagnosis.

**Figure 1.**
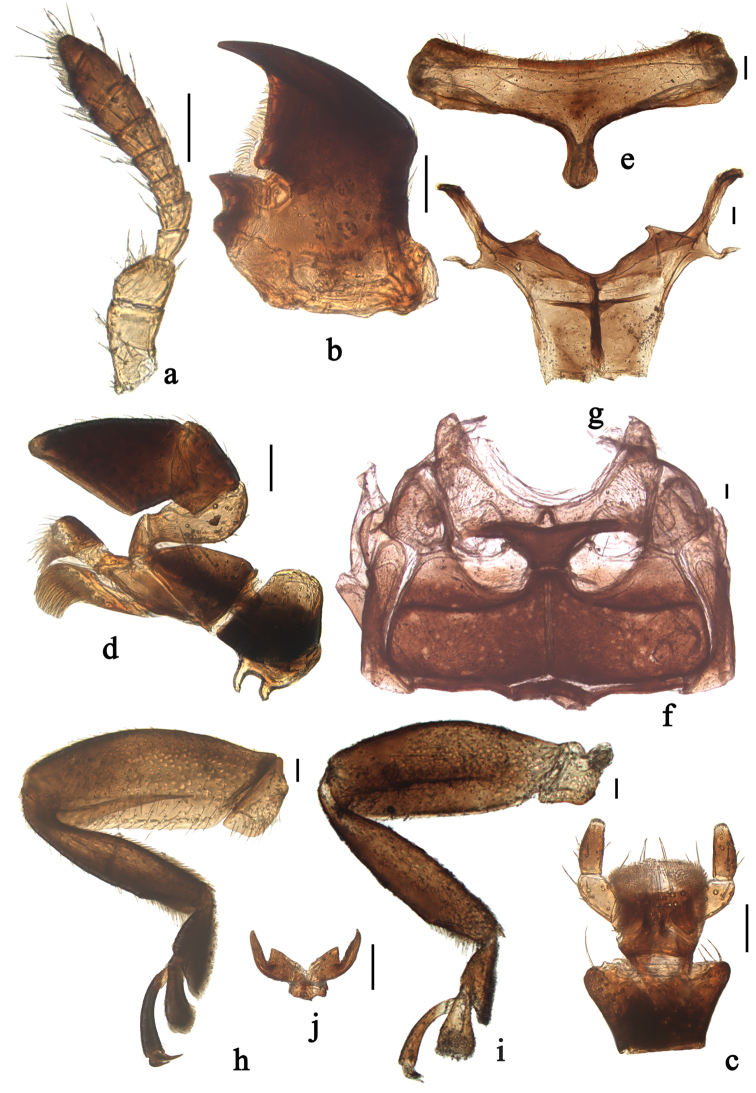
*Xanthocorus nigromarginatus* (Miyatake, 1970). **a** antenna **b** mandible **c** labium **d** maxilla **e** prosternum **f** meso- and metaventrite **g** metendosternite **h** front leg **i** hind leg **j** tarsal claws. Scale bars: 0.1 mm.

**Figure 2.**
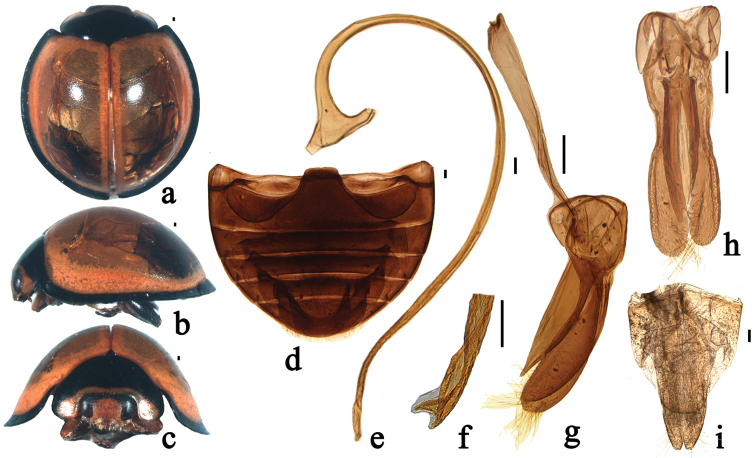
*Xanthocorus nigromarginatus* (Miyatake, 1970). **a** dorsal view **b** lateral view **c** anterior view **d** abdomen **e** penis **f** apex of penis **g** tegmen, lateral view **h** tegmen, ventral view **i** female genitalia: ovipositor. Scale bars: 0.1 mm.

**Figure 3.**
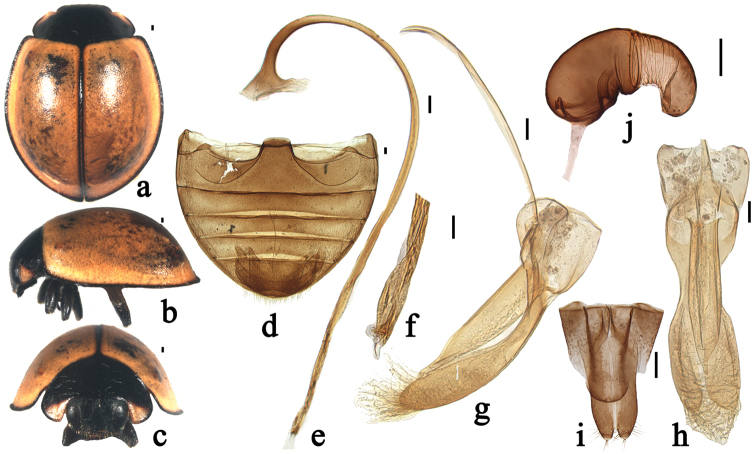
*Xanthocorus nigrosuturalis* sp. n. **a** dorsal view **b** lateral view **c** anterior view **d** abdomen **e** penis **f** apex of penis **g** tegmen, lateral view **h** tegmen, ventral view **i**–**j** female genitalia: **i** ovipositor **j** spermatheca. Scale bars: 0.1 mm.

**Figure 4.**
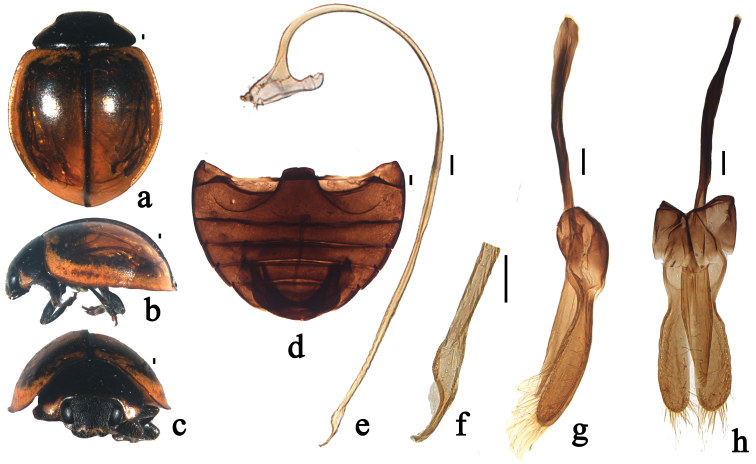
*Xanthocorus mucronatus* sp. n. **a** dorsal view **b** lateral view **c** anterior view **d** abdomen **e** penis **f** apex of penis **g** tegmen, lateral view **h** tegmen, ventral view. Scale bars: 0.1 mm.

The genus *Xanthocorus* can be distinguished from other genera of the tribe Chilocorini by the following combination of characters: antenna composed of 10 antennomeres, relatively slender, terminal antennomere very small and inserted in antennomere 9 (Fig. 1a); pronotal basal margin without bordering line; prosternal process narrow, without carinae (Fig. 1e); elytral epipleura distinctly oblique and without foveae; abdominal postcoxal lines semicircular, incomplete laterally (Figs 2d, 3d, 4d); front tibiae without apical spurs, mid and hind tibiae with two apical spurs (Figs 1h, 1i); tarsal claw with basal tooth (Fig. 1j).

#### Description.

Body broadly oval to almost circular in outline, moderately convex. Dorsum glabrous.

Head relatively large, 0.50–0.58 times pronotal width, covered with short, greyish pubescence; antenna composed of 10 antennomeres, relatively slender, scape and pedicel stout, of the same width, and pedicel distinctly shorter than scape, antennomeres 3–8 gradually broadening, antennomere 9 slightly narrower than 8, and terminal antennomere short, partially embedded in antennomere 9 (Fig. 1a). Mandible unidentate, prostheca distinct, lateral margin of mandible strongly curved (Fig. 1b). Terminal maxillary palpomere broadening apically, apical margin strongly obliquely truncate (Fig. 1d). Terminal labial palpomere stout with rounded apex (Fig. 1c).

Prothorax descending anteriorly. Base of pronotum and elytra not contiguous all along their length. Basal margin of pronotum without bordering line. Prosternum T-shaped, in front of coxae distinctly longer than basal width of prosternal process. Prosternal process narrow, parallel sided, without carinae (Fig. 1e). Mesoventrite approximately trapezoidal, mesal surface with emarginate fossa for receiving apex of prosternal process. Mesoventral process narrow; meso-metaventral process narrow, junction arcuate anteriorly, with visible suture (Fig. 1f). Metendosternite stalk as long as broad (Fig. 1g). Scutellum small,triangular. Elytra distinctly wider than pronotum at base, surface finely or coarsely punctate. Elytral epipleura distinctly oblique and without foveae. Abdomen with five ventrites in female and six ventrites in male. Abdominal postcoxal lines recurved, incomplete laterally. Front tibiae without apical spurs, mid and hind tibiae with two apical spurs (Fig. 1h, 1i); tarsal claw with basal tooth (Fig. 1j).

#### Key to species of the genus *Xanthocorus* from China

**Table:** 

1	Body broadly oval, elytra yellow or brownish yellow, suture black	**2**
–	Body almost circular, elytra yellow with only lateral margins black (Fig. 2a)	***Xanthocorus nigromarginatus***
2	Elytra yellow, with lateral and anterior margins, and suture black (Fig. 3a), penis with blunt and bifurcate apex (Fig. 3f)	***Xanthocorus nigrosuturalis* sp. n.**
–	Elytra brownish yellow, only elytral suture black (Fig. 4a), penis with pointed apex (Fig. 4f)	***Xanthocorus mucronatus* sp. n.**

### Xanthocorus nigromarginatus

Taxon classificationAnimaliaColeopteraCoccinellidae

(Miyatake, 1970)

**Figure 5.**
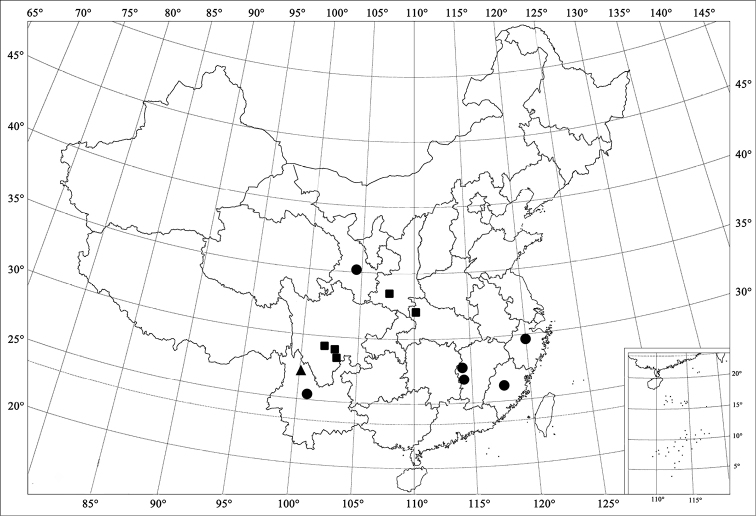
Distribution map. (●) *Xanthocorus nigromarginatus* (Miyatake, 1970); (■) *Xanthocorus nigrosuturalis* sp. n.; (▲) *Xanthocorus mucronatus* sp. n.

Figs 1a–j , 2a–i , 5

Exochomus (Xanthocorus) nigromarginatus Miyatake, 1970: 312; Pang and Mao 1979: 76; Cao et al. 1992: 152; Pang et al. 2004: 316; Ren et al. 2009: 136.
Xanthocorus nigromarginatus : Kovář, 1997: 24.

#### Diagnosis.

This species can be distinguished from other species of *Xanthocorus* by the following combination of characters: body almost circular, pronotum black with anterior angles and anterior margin yellow; elytra yellow, only elytral lateral margins of elytra black (Fig. 2a–c); apex of penis bifurcate, penis guide slightly asymmetrical, parameres stout (Fig. 2e–h).

#### Description.

TL: 5.53–5.92 mm, TW: 5.11–5.30 mm, TH: 2.80–2.88 mm, TL/TW: 1.08–1.12, PL/PW: 0.49–0.50, EL/EW: 0.97–0.99.

Body almost circular, moderately convex. Head yellow with black vertex in male, entirely black in female. Mouthparts and antennae brown, sparsely covered with short, greyish pubescence. Pronotum black, only anterior angles and anterior margin yellow. Scutellum black. Elytra yellow, only elytral bead black (Fig. 2a–c). Underside black except inner part of elytral epipleura yellow and abdominal ventrites brownish black, sparsely covered with short, greyish pubescence.

Head relatively large, 0.50 times pronotal width, punctation on frons large and densely distributed, 0.5–1.5 diameters apart, surface polished between punctation. Eyes approximately oval, densely faceted, interocular distance 0.54 times head width (Fig. 2c). Pronotum 0.54 times elytral width, pronotal punctation large and densely distributed, smaller than those on head, 1.0–2.0 diameters apart, surface polished between punctation. Punctation on elytra moderately large and densely distributed, 1.0–2.0 diameters apart, similar to those on pronotum. Prosternal process narrow with sides parallel. Posterior margin of abdominal ventrite 5 and 6 slightly emarginate medially in male (Fig. 2d).

Male genitalia: penis slender, penis capsule with short outer and inner arms, apex of penis bifurcate with membranous appendage (Fig. 2e–f). Tegmen stout with penis guide slightly asymmetrical in ventral view and widest at base with sides parallel from base to 1/2 length, then gradually converging to blunt apex in lateral view. Parameres stout, distinctly longer than penis guide, densely covered with long setae at inner surfaces and apices with group of long setae in lateral view (Fig. 2g–h).

Female genitalia: coxites distinctly elongate, approximately triangular (Fig. 2i).

#### Material examined.

China: Jiangxi Prov: 2 males and 1 female, Luofu village, Jinggangshan County, [26°65.41'N; 114°22.49'E], ca 763m, 18.ix.2004, Wang XM leg; 1 female, Jingzhu Mountain, Jinggangshan County, [26°37.95'N; 114°08.98'E], ca 1142m, 22.ix.2004, Wang XM leg.

#### Distribution.

China (Gansu, Zhejiang, Jiangxi, Fujian, Yunnan) (Fig. 5).

#### Remarks.

The Chinese specimens of this species studied were identical with the illustrations and descriptions of adult and male genitalia given by Miyatake (1970).

### Xanthocorus nigrosuturalis

Taxon classificationAnimaliaColeopteraCoccinellidae

Li & Ren sp. n.

http://zoobank.org/720BAC11-200D-408E-8D30-B68B2FD92B95

Figs 3a–j , 5

#### Diagnosis.

This species resembles *Xanthocorus nigromarginatus*, but can be distinguished from it by having slightly elongate body; black pronotum with only anterior angles yellow; elytra yellow with black elytral lateral margin, anterior margin and suture (Fig. 3a–c); slender parameres (Fig. 3g). In *Xanthocorus nigromarginatus*, body almost circular, pronotum black except anterior angles and anterior margin yellow; elytra yellow, only elytral lateral margin black (Fig. 2a–c); parameres stout (Fig. 2g).

#### Description.

TL: 4.16–5.79 mm, TW: 3.35–4.81 mm, TH: 1.83–2.77 mm, TL/TW: 1.20–1.24, PL/PW: 0.47–0.51, EL/EW: 1.04–1.05.

Body broadly oval, moderately convex. Head and mouthparts black. Antenna brownish yellow, sparsely covered with short, greyish pubescence. Pronotum black with anterior angles yellow. Scutellum black. Elytra yellow with black elytral bead, anterior margin and suture (Fig. 3a–c). Underside black except inner margins of elytral epipleura yellow. Abdominal ventrites brown, sparsely covered with short, greyish pubescence.

Head relatively large, 0.56 times pronotal width, punctation on frons large and densely distributed, 1.0–1.5 diameters apart, surface polished between punctation. Eyes approximately oval, densely faceted, interocular distance 0.51 times head width (Fig. 3c). Pronotum 0.55 times elytral width, pronotal punctation large and moderately densely distributed, smaller than those on head, 2.0–3.0 diameters apart, surface polished between punctation. Punctation on elytra moderately large and moderately densely distributed, 2.0–3.0 diameters apart, similar to those on pronotum. Prosternal process narrow with parallel sided. Posterior margin of male abdominal ventrite 5 distinctly emarginate and ventrite 6 slightly emarginate medially (Fig. 3d).

Male genitalia: penis slender, penis capsule with short outer and inner arms. Apex of penis bifurcate with membranous appendage (Fig. 3e–f). Tegmen stout with penis guide distinctly asymmetrical in ventral view and with sides parallel from base to 2/3 length, then abruptly converging to blunt apex in lateral view. Parameres slender, strongly constricted at base, expanded toward apex, distinctly longer than penis guide, densely covered with long setae at inner surfaces and apices with group of long setae in lateral view (Fig. 3g–h).

Female genitalia: coxites distinctly elongate, approximately triangular (Fig. 3i). Spermatheca C-shaped, cornu without appendage (Fig. 3j).

#### Types.

Holotype. male, China: Shannxi Prov: Daguping and Xihe Conservation Station, Foping National Nature Reserve, No. SCAU (E) 11475, [33°35.57'N; 107°50.25'E], ca 1428m, 22.vii.2009, Wang XM leg (SCAU). Paratypes. 1 male and 1 female with same data as holotype (1 female in SCAU, 1 male in IOZ); Sichuan Prov: 7 females, Mamize village, Leibo County, [28°25.02'N; 103°36.13'E], ca 2600m, 19–20.ix.2007, Liang JB leg (SCAU); 2 females, Dafengding National Nature Reserve, Meigu County, [28°60.77'N; 103°23.72'E], ca 2400m, 21.ix.2007, Chen XS leg (SCAU); 2 males and 1 female, Liziping National Nature Reserve, Shimian County, [29°09.89'N; 102°33.84'E], ca 2000m, 26–27.ix.2007, Chen XS leg (2 males in SCAU, 1 female in IOZ). Hubei Prov: 1 male and 1 female, Banqiao Conservation Station, Shennongjia National Nature Reserve, [31°45.90'N; 110°37.02'E], ca 1170m, 21–24.vii.2007, Wang XM leg (SCAU); 4 females, Guanmenshan Scenic Spot, Shennongjia National Nature Reserve, [31°42.94'N; 110°36.53'E], ca 1260m, 2.viiiI.2007, Chen XS leg (SCAU).

#### Distribution.

China (Hubei, Sichuan, Shannxi) (Fig. 5).

#### Etymology.

The species name is derived from Latin and refers to the black elytral suture.

### Xanthocorus mucronatus

Taxon classificationAnimaliaColeopteraCoccinellidae

Li & Ren sp. n.

http://zoobank.org/190749C1-140A-438F-877B-2011BCCB6D7D

Figs 4a–h , 5

#### Diagnosis.

This species is similar to *Xanthocorus nigrosuturalis* sp. n., but can be distinguished from it by having lateral margins of elytra yellow (Fig. 4a) and apex of penis pointed (Fig. 4f). In *Xanthocorus nigrosuturalis* sp. n., elytral lateral margin is black (Fig. 3a) and penis has blunt and bifurcate apex (Fig. 3f).

#### Description.

TL: 3.75 mm, TW: 2.98 mm, TH: 1.62 mm, TL/TW: 1.27, PL/PW: 0.49, EL/EW: 1.01.

Body broadly oval, moderately convex. Head black except sides of clypeus yellow. Mouthparts black. Antenna brown, sparsely covered with short, greyish pubescence. Pronotum black with anterior angles yellow. Scutellum black. Elytra yellow with black suture (Fig. 4a–c). Underside black except elytral epipleura yellow, sparsely covered with short, greyish pubescence.

Head relatively large, 0.58 times pronotal width, punctation on frons fine and moderately densely distributed, 2.0–3.0 diameters apart, surface polished between punctation. Eyes approximately oval, densely faceted, interocular distance 0.55 times head width (Fig. 4c). Pronotum 0.55 times elytral width, pronotal punctation fine and sparsely distributed, smaller than those on head, 3.0–4.0 diameters apart, surface polished between punctation. Punctation on elytra moderately fine and sparsely distributed, 2.0–3.0 diameters apart, similar to those on pronotum. Prosternal process narrow with sides parallel. Posterior margin of abdominal ventrite 5 and 6 distinctly emarginated medially in male (Fig. 4d).

Male genitalia: penis slender, penis capsule with short outer and inner arms. Apex of penis strongly narrow with membranous appendage (Fig. 4e–f). Tegmen stout with penis guide with parallel sided from base to 1/2 length, then gradually converging to blunt apex in ventral view; in lateral view, penis guide widest at base, gradually constricted to apex. Parameres strongly constricted at base and expanded toward apex, longer than penis guide, densely covered with long setae at the inner sides and distal end with a group of long setae in lateral view (Fig. 4g–h).

Female genitalia: unknown.

#### Types.

Holotype. male, **China: Yunnan Prov**: Shangri-La, No. 20051215051, [27°90.22'N; 99°63.37'E], ca 3450m, 3.ix.2005, Wang XM leg (SCAU).

#### Distribution.

China (Yunnan) (Fig. 5).

#### Etymology.

The species name is derived from Latin and refers to the pointed apex of penis.

## Supplementary Material

XML Treatment for Xanthocorus

XML Treatment for Xanthocorus nigromarginatus

XML Treatment for Xanthocorus nigrosuturalis

XML Treatment for Xanthocorus mucronatus
